# The Efficacy and Safety of Traditional Chinese Medicine Tonifying-Shen (Kidney) Principle for Primary Osteoporosis: A Systematic Review and Meta-Analysis of Randomized Controlled Trials

**DOI:** 10.1155/2020/5687421

**Published:** 2020-10-06

**Authors:** Junquan Liang, Fengyi Wang, Jiajia Huang, Yunxiang Xu, Guizhen Chen

**Affiliations:** ^1^Clinical Medical School of Acupuncture, Moxibustion and Rehabilitation, Guangzhou University of Chinese Medicine, Guangzhou 510405, Guangdong, China; ^2^The Bao'an District TCM Hospital, The Affiliated Hospital of Guangzhou University of Chinese Medicine, Guangzhou University of Chinese Medicine, Shenzhen 518101, Guangdong, China

## Abstract

**Objective:**

This study aimed to appraise the efficacy and safety of the tonifying-Shen (kidney) principle (TS (TK) principle) for primary osteoporosis (POP).

**Methods:**

Randomized controlled clinical trials (RCTs) using the TS (TK) principle for POP were searched from eight electronic databases to search for relevant literature that was published from the initiation to September 2019. Two reviewers performed study selection, data extraction, data synthesis, and quality assessment independently. Review Manager 5.3 software was used to assess the risk of bias and conduct the data synthesis. We assessed the quality of evidence for outcomes by using the Grading of Recommendations Assessment, Development, and Evaluation (GRADE) system.

**Results:**

Thirty-six studies with 3617 participants were included. Meta-analysis showed a consistently superior effect of the TS (TK) principle combined with conventional Western medicine (CWM) in terms of total effectiveness rates (RR = 1.28; 95% CI (1.23, 1.33); *P* < 0.00001), BMD of the lumbar spine (SMD = 0.71; 95% CI (0.47, 0.95); *P* < 0.00001) and proximal femur (SMD = 0.94; 95% CI (0.49, 1.38); *P* < 0.00001), TCM symptom integral (SMD = −1.23; 95% CI (−1.43, −1.02); *P* < 0.00001), and VAS scores (SMD = −3.88; 95% CI (−5.29, −2.46); *P* < 0.00001), when compared to using CWM alone and with significant differences. Besides, in respect of adverse effects, it showed no significant statistical difference between the experimental and control groups, RR = 0.99 and 95% CI (0.65, 1.51), *P*=0.97.

**Conclusion:**

Our meta-analysis provides promising evidence to suggest that using the TS (TK) principle combined with CWM for POP is more effective than using CWM alone. Also, both of them are safe and reliable for POP.

## 1. Introduction

Primary osteoporosis (POP), also called “a silent disease,” is one of the pandemic public health issues that seriously threaten people's health all over the world [1]. Theoretically, POP and secondary osteoporosis belong to the field of osteoporosis (OP). POP includes two major types: postmenopausal osteoporosis (PMOP) and age-related or senile osteoporosis [2]. POP is characterized by decreased bone mass and degenerated bone microstructure, which contributes to a high risk of bone fragility and fracture [3, 4]. It is estimated that the number of individuals aged 50 years or over at high risk of osteoporotic fracture worldwide was at 158 million in 2010 and is set to double by 2040 [5]. In China, because of the largest population and the increasing proportion of elderly people, osteoporosis has become a severe challenge to the Chinese family, society, and government [6, 7]. Therefore, an effective prevention and treatment method is urgently needed for POP. Management of POP includes nonpharmacologic treatment and pharmacologic treatment. Conventional Western medicines (CWM) including antiresorptive or anabolic are widely used in the treatment of POP [8–10]. However, due to adverse effects and risk of cancer, the use of CWM as long-term treatment is limited [11].

It is well documented that traditional Chinese medicine (TCM) is commonly combined with pharmacotherapy for POP in China [12–14]. In the theory of TCM, there is no particular disease named POP. According to the clinical symptoms, POP belongs to the TCM category of “Guwei (flaccidity of bones),” “Guku (dryness of bones),” “Guji (disease of bones),” “Gukong (depletion of bones), and Gubi (impediment of bones)” [15]. “Shen (kidney) dominating the bone” is the most fundamental theory in TCM. Shen essence (kidney essence) is closely related to bone physiology and pathology. The sufficient Shen essence can keep the bone healthy and strong. On the contrary, the deficiency of Shen essence will lead to a series of bone-related symptoms. Besides, “liver controlling tendon,” is involved in bone health. Also, the spleen and stomach are considered as the postnatal foundations of organisms and are the material sources of bone growth. Therefore, the tonifying-Shen (kidney) principle (TS (TK) principle) mainly includes bushen huoxue, bushen zhuanggu, bushen jianpi, and buyi ganshen, which are widely used in combination with other TCM therapies or CWM for the treatment of POP. And, this significant principle has its essential meaning in curing POP [16].

In recent years, numerous meta-analyses were carried out to investigate the efficacy of acupuncture or Chinese herbal medicine for POP [17]. However, there was still no sufficient evidence to draw definitive conclusions as most studies were not comparative analysis aiming at the effect of the TS (TK) principle for POP. Besides, most of the meta-analyses did not explore the safety of particular interventions. Therefore, the purpose of this study was to systematically identify available randomized clinical trials (RCTs) using the TS (TK) principle combined with CWM for POP to appraise its efficacy and safety.

## 2. Materials and Methods

### 2.1. Study Registration

The study has been registered in PROSPERO (registration number: CRD42020151768). The review reporting was conducted in compliance with the preferred reporting items for systematic reviews and meta-analyses (PRISMA) statement guidelines.

### 2.2. Study Design

#### 2.2.1. Inclusion Criteria

*(1) Types of Participants and Interventions*. All RCTs that were reporting the application of the TS (TK) principle combined with CWM for POP were included. The interventions of these studies must include the TS (TK) principle in the experimental group. Studies must be published in English or Chinese language. TS (TK) principle mainly includes bushen huoxue, bushen zhuanggu, bushen jianpi, and buyi ganshen. Specific treatment methods include traditional Chinese herbal medicine, acupuncture and moxibustion combined with traditional Chinese herbal medicine, acupoint catgut embedding, and acupoint injection. POP patients were included. There is no restriction on gender, race, ethnicity, or nation. Patients in the treatment group were given the TS (TK) principle combined with CWM, while patients in the control group were given CWM alone. The dosages and courses were not limited in our studies.

*(2) Types of Outcome Measures*. The primary outcomes included effectiveness rate and bone mineral density (BMD) of the lumbar spine and proximal femur (femoral neck or total hip). The secondary outcomes consisted of VAS scores and TCM symptom integral. Besides, adverse events were also assessed as a safety measurement.

#### 2.2.2. Exclusion Criteria

Studies with the following characteristics were excluded: irrelevant to TS (TK) principle studies; studies without consistent diagnostic criteria or relevant outcome indicators; non-English or Chinese-language articles; duplicate reports or the data cannot be extracted; case reports, animal experiences, qualitative studies, comments, or review articles.

### 2.3. Literature Search Strategy

We searched four international electronic databases (PubMed, Cochrane Library, EMBASE, and Web of Science) and four Chinese electronic databases (CNKI, VIP, Wanfang, and CBM) from their initiation to September 2019 to collect for relevant literature. The literature search was constructed around search terms for TS (TK) principle, POP, and randomized controlled trials and adapted for each database as necessary. The references of the included studies were also screened for further material for inclusion. The detailed search strategy for PubMed is in Table 1. Search strategies were also used for other electronic databases.

### 2.4. Study Selection and Data Extraction

As a first step in the data handling process, titles and abstracts of all studies retrieved by the search strategies were screened for relevance, and all those that were clearly irrelevant have been discarded.

As a second step, two review team members (Junquan Liang and Fengyi Wang) independently assessed the eligibility of the studies by using the predefined inclusion and exclusion criteria. Besides, for the studies that meet the inclusion criteria, the whole article was read by reviewers to ensure that the entire study met the criteria and was prepared to extract relevant information. The disagreements on whether including a specific study or not were resolved by discussion between the reviewers. The lacking information was requested by contacting the writer of the original article.

The information extracted by the two independent review team members included the following: study setting, population study, participant demographics and baseline characteristics, details of the intervention and control conditions, study methodology, outcomes and treatment periods, information for the assessment of the risk of bias. The discrepancies were identified and resolved through discussion (with a third author where necessary). Missing data were requested from the study authors.

### 2.5. Risk of Bias Assessment

There were two reviewers involved in the quality assessment process, and any major disagreements were resolved by discussion to define the final set of included studies.

Two independent reviewers assessed the risk of bias by considering the following characteristics: randomization sequence generation, treatment allocation concealment, blinding method, completeness of outcome data, selective outcome reporting, and other sources of bias. Besides, the Cochrane Collaboration's risk of bias assessment tool was used to assess the quality of the individual included studies.

### 2.6. Data Synthesis

Review Manager 5.3 software was used to carry out the quantitative synthesis. Mean difference (MD) or standardized mean difference (SMD) was used for continuous data. Risk ratio (RR) was used for the analysis of dichotomous data. Both were given a 95% confidence interval (CI). In the case of homogeneous data (*I*^2^ ≤ 50%, *P* > 0.10), the fixed-effect model was adopted for the meta-analysis. Otherwise, the sources of heterogeneity were further analyzed. After excluding the influence of marked clinical heterogeneity, a random-effect model was adopted to perform the meta-analysis. Sensitivity and bias risk analyses were also performed.

#### 2.6.1. Analysis of Subgroups

We performed some planned subgroup analysis: different specific therapies (bushen huoxue, bushen zhuanggu, bushen jianpi, and buyi ganshen) included in the TS (TK) principle, different kinds of treatment methods (traditional Chinese herbal medicine, acupuncture and moxibustion combined with traditional Chinese herbal medicine, acupoint catgut embedding, and acupoint injection), different parts of BMD examination (lumbar spine and proximal femur (femoral neck or total hip)), and different treatment periods of the TS (TK) principle (≤3 months, 3–6 months, and >6 months).

#### 2.6.2. Sensitivity Analysis and Reporting Bias Analysis

Sensitivity analysis was carried out to identify the robustness and stability of pooled outcome results by removing the low-quality studies. We have performed a funnel plot of the primary outcome (effectiveness rates of different treatment methods included in the tonifying-Shen (kidney) principle) to evaluate the reporting bias.

### 2.7. Quality of Evidence

We assessed the quality of evidence for outcomes by using the Grading of Recommendations Assessment, Development, and Evaluation (GRADE) system [18].

## 3. Results

### 3.1. Study Description and Participants

**Figure 1:**
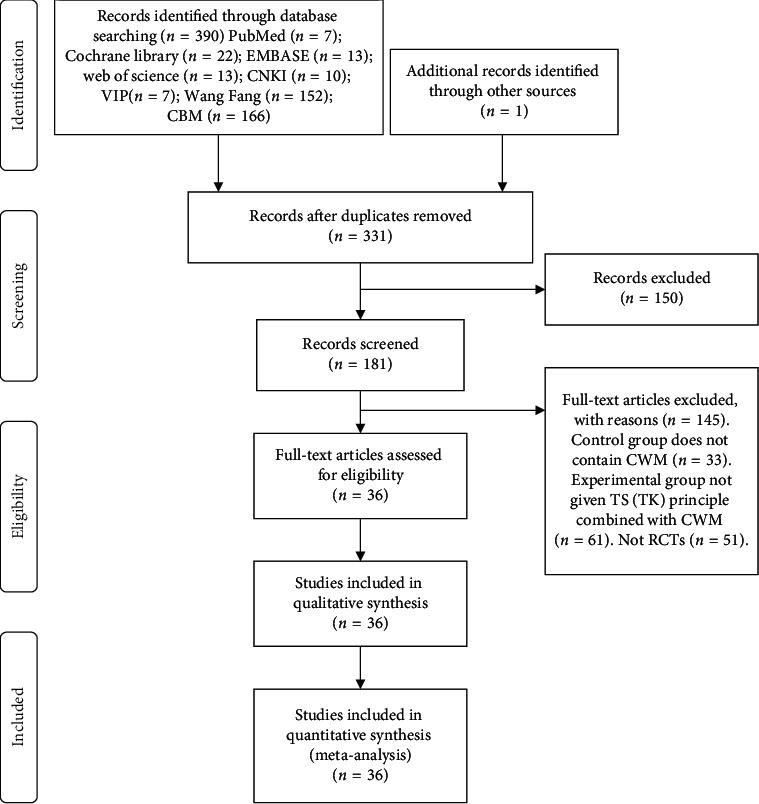
The flowchart of the selection procedure.

We obtained 390 relevant studies through preliminary searches. After multiple filtering steps, 36 RCTs with a total of 3617 participants were ultimately included in this systematic review. The flowchart of all study selection procedures is shown in Figure 1.

The 36 included studies involved 3617 participants. Among these studies, apart from combined with CWM, 23 studies reported using TCM herbal medicine [19–41], 5 studies reported using acupuncture and moxibustion combined with traditional Chinese herbal medicine [42–46], 9 studies reported using acupoint catgut embedding [25, 26, 40, 47–52], and 2 studies reported using acupoint injection [53, 54]. Besides, 3 studies divided their experimental group into two groups, respectively (acupoint catgut embedding group and TCM herbal medicine group) [25, 26, 40]. The detailed characteristics of the included studies are shown in Table 2.

### 3.2. Risk of Bias Assessment

**Figure 2:**
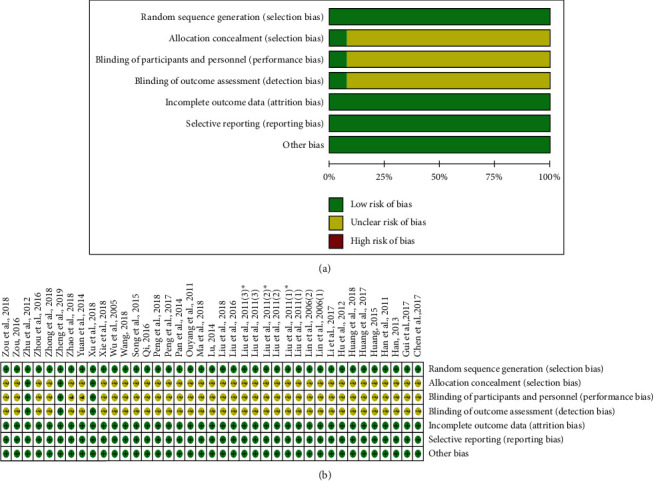
Risk of bias assessment. Notes: the experimental group of Liu 2011 (1) was divided into 2 groups, respectively: Liu 2011 (1) and Liu 2011 (1)^*∗*^; the experimental group of Liu 2011 (2) was divided into 2 groups, respectively: Liu 2011 (2) and Liu 2011 (2)^*∗*^; the experimental group of Liu 2011 (3) was divided into 2 groups, respectively: Liu 2011 (3) and Liu 2011 (3)^*∗*^.

We used the Cochrane Collaboration's risk of bias assessment tool to assess the quality of the included studies. Firstly, all studies reported the method of randomization, and 30 studies described the method of generating a randomization number table [19–30, 32, 34, 35, 37–42, 44, 46, 48–54]. The remaining methods to achieve the sequence generation process include the following: drawing opaque envelope randomly [43], using Doll's clinical case random table [47], drawing of lots, [36, 45], and tossing coins [31, 33]. Secondly, there were only 3 studies which achieved allocation concealment [19, 20, 43]. Thirdly, 3 studies were assessed as appropriate double-blinding of participants and provided detailed information for double-blinding during treatment as well as an outcome assessment [19, 20, 43]. None of the studies reported any incomplete outcome data (Table 3 and Figure 2).

### 3.3. Meta-Analysis

#### 3.3.1. Effectiveness Rates of Different Treatment Methods Included in TS (TK) Principle

**Figure 3:**
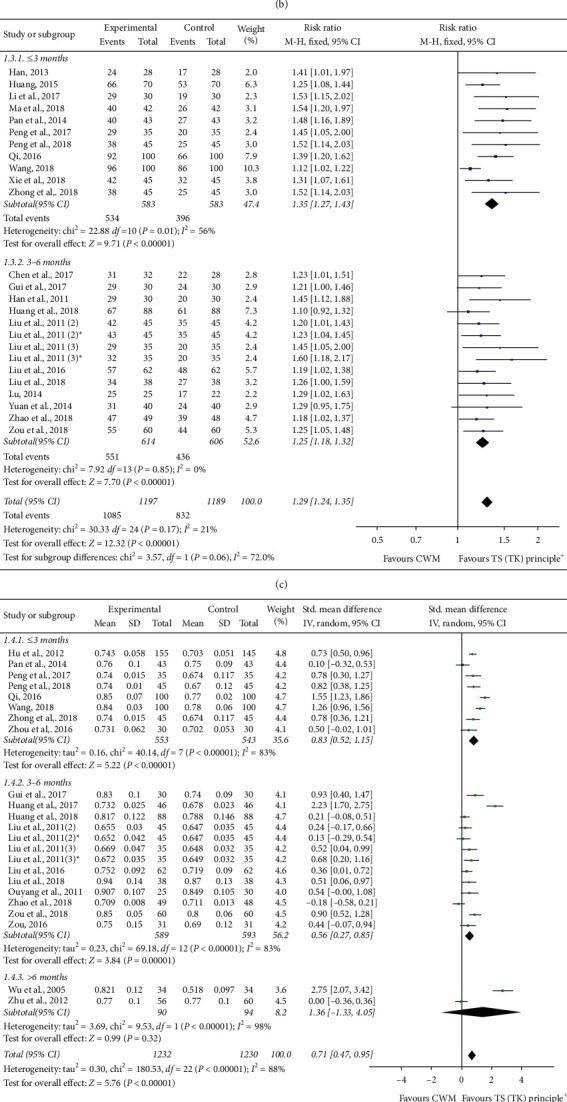
(a) Effectiveness rates of different treatment methods included in the TS (TK) principle. (b) Effectiveness rates of different specific therapies included in TCM herbal medicine. (c) Effectiveness rates of different treatment periods. (d) BMD (lumbar spine) of different treatment periods. (e) BMD (proximal femur (femoral neck or total hip)) of different treatment periods. (f) Adverse effects. (g) TCM symptom integral. (h) VAS scores. Notes: the experimental group of Liu 2011 (2) was divided into 2 groups, respectively: Liu 2011 (2) and Liu 2011 (2)^*∗*^; the experimental group of Liu 2011 (3) was divided into 2 groups, respectively: Liu 2011 (3) and Liu 2011 (3)^*∗*^.

Twenty-five RCTs reported effectiveness rates of different treatment methods included in the TS (TK) principle [21–23, 25, 27, 28, 30, 31, 33, 34, 36–42, 44–50, 53]. Sixteen RCTs reported effectiveness rates of TCM herbal medicine [21–23, 25, 27, 28, 30, 31, 33, 34, 36–41], and there was low statistical heterogeneity among studies (chi^2^ = 19.09, *P*=0.21; *I*^2^ = 21%). Therefore, the fixed-effect model was applied to calculate the combined RR and 95% CI as 1.24 (1.19, 1.30), *P* < 0.00001, indicating a statistically significant difference between TCM herbal medicine combined with CWM and CWM alone. This result suggests that TCM herbal medicine combined with CWM in the treatment of POP can significantly improve clinical efficacy when compared with using CWM alone. Three studies reported effectiveness rates of acupuncture and moxibustion combined with TCM herbal medicine [42, 44, 45]. The result showed that there was no statistical heterogeneity among studies (chi^2^ = 3.94, *P*=0.14; *I*^2^ = 49%), so we adopted a fixed-effect model to calculate the combined RR and 95% CI as 1.34 (1.19, 1.51), *P* < 0.00001, indicating a statistically significant difference between the experimental group and the control group. This result suggests that, in respect of effectiveness rates, using acupuncture and moxibustion combined with TCM herbal medicine plus CWM for POP was better than using CWM alone. There are 6 studies which reported effectiveness rates of acupoint catgut embedding [25, 40, 47–50]. The heterogeneity was not detected among studies (chi^2^ = 4.53, *P*=0.48; *I*^2^ = 0%), so a fixed-effect model was used to calculate the combined RR and 95% CI as 1.42 (1.27, 1.58), *P* < 0.00001, indicating a statistically significant difference between acupoint catgut embedding combined with the CWM group and the CWM alone group. This result shows that the effectiveness rates of catgut embedding combined with CWM for POP were better than using CWM alone. There is only 1 study which reported effectiveness rates of acupoint injection [53]. The combined RR and 95% CI was 1.25 (1.05, 1.48), *P* < 0.00001, indicating the difference between acupoint injection combined with the CWM group and the CWM alone group. Owing to the small sample size, this result would show that the effectiveness rates of acupoint injection combined with CWM for POP were better than using CWM alone. All in all, the pooled data showed that different treatment methods included in the TS (TK) principle combined with CWM were more effective than using CWM alone in improving effectiveness rates, with significant differences (RR = 1.28; 95% CI (1.23, 1.33); *P* < 0.00001) (Figure 3(a)).

#### 3.3.2. Effectiveness Rates of Different Specific Therapies Included in TCM Herbal Medicine

There were 16 studies which reported effectiveness rates of different specific therapies included in TCM herbal medicine [21–23, 25, 27, 28, 30, 31, 33, 34, 36–41]. Five studies reported the effectiveness rates of bushen huoxue therapy [23, 36–39], three studies reported the effectiveness rates of bushen zhuanggu therapy [27, 31, 33], three studies reported the effectiveness rates of bushen jianpi therapy [22, 28, 30], and five studies reported the effectiveness rates of buyi ganshen therapy [21, 25, 34, 40, 41]. The heterogeneity among these studies was chi^2^ = 3.07, *P*=0.55; *I*^2^ = 0%, chi^2^ = 1.05, *P*=0.59; *I*^2^ = 0%, chi^2^ = 1.16, *P*=0.56; *I*^2^ = 0%, and chi^2^ = 3.02, *P*=0.55; *I*^2^ = 0%, respectively. Therefore, the fixed-effect model was applied to calculate the combined RR and 95% CI. After calculating, the combined RR and 95% CI was 1.18 (1.11, 1.24); *P* < 0.00001, 1.36 (1.21, 1.53); *P* < 0.00001, 1.34 (1.15, 1.57); *P*=0.0002, and 1.21 (1.11, 1.32); *P* < 0.00001, respectively, indicating a statistically significant difference between the experimental group and the control group. This result suggests that bushen huoxue, bushen zhuanggu, bushen jianpi, and buyi ganshen TCM herbal medicine combined with CWM in the treatment of POP can significantly improve clinical efficacy when compared with using CWM alone. The combined data showed that different specific therapies included in TCM herbal medicine combined with CWM were more effective than using CWM alone in improving effectiveness rates, with significant differences (RR = 1.23; 95% CI (1.18, 1.29); *P* < 0.00001) (Figure 3(b)).

#### 3.3.3. Effectiveness Rates of Different Treatment Periods

Twenty-three literature studies reported effectiveness rates of different treatment periods [21–23, 26–28, 30, 31, 33, 34, 37–42, 44, 45, 47–50, 53]. Eleven literature studies reported treatment periods of less than 3 months [23, 27, 28, 37, 38, 41, 44, 45, 48–50]. However, there was high statistical heterogeneity among studies (chi^2^ = 22.88, *P*=0.01; *I*^2^ = 56%). A sensitivity analysis was performed to identify the source of heterogeneity. By removing one trial [38], no heterogeneity was detected (chi^2^ = 4.88, *P*=0.84; *I*^2^ = 0%). We confirmed the accuracy of the data without publication bias after contacting the author. So, we adopted a random-effect model to calculate the combined RR and 95% CI as 1.35 (1.27, 1.43), *P* < 0.00001, indicating a statistically significant difference between the TS (TK) principle combined with the CWM group and the CWM alone group. It is suggested that using the TS (TK) principle combined with CWM for POP was better than using CWM alone in improving effectiveness rates when the treatment periods were less than 3 months. Twelve literature studies reported treatment periods of three to six months [21, 22, 25, 30, 31, 34, 39, 40, 42, 44, 47, 53]. There was no statistical heterogeneity among studies (chi^2^ = 7.92, *P*=0.85; *I*^2^ = 0%). Therefore, the fixed-effect model was applied to calculate the combined RR and 95% CI as 1.25 (1.18, 1.32), *P* < 0.00001, indicating a statistically significant difference between the TS (TK) principle combined with the CWM group and the CWM alone group. It is suggested that using the TS (TK) principle combined with CWM for POP was better than using CWM alone in improving effectiveness rates when the treatment periods were three to six months. The pooled data showed that different treatment periods combined with CWM were more effective than using CWM alone in improving effectiveness rates, with significant differences (RR = 1.29; 95% CI (1.24, 1.35); *P* < 0.00001) (Figure 3(c)).

#### 3.3.4. BMD (Lumbar Spine) of Different Treatment Periods

Twenty-one studies reported BMD (lumbar spine) of different treatment periods [20, 21, 24, 25, 27, 29, 30, 32, 34, 35, 37–40, 42, 46, 48–50, 53, 54]. There were 8 studies which reported BMD (lumbar spine) of less than 3 months [27, 29, 37, 38, 48–50, 54], and 11 studies reported BMD (lumbar spine) of three to six months [21, 25, 30, 32, 34, 35, 39, 40, 42, 46, 53]. However, we detected high statistical heterogeneity among studies, chi^2^ = 40.14, *P* < 0.00001; *I*^2^ = 83% and chi^2^ = 69.18, *P* < 0.00001; *I*^2^ = 83%. The source of heterogeneity may be related to different treatment methods. Therefore, we adopted the random-effect model, and meta-analysis showed that there was a significant difference between the experimental and control groups (SMD = 0.83; 95% CI (0.52, 1.15); *P* < 0.00001 and SMD = 0.56; 95% CI (0.27, 0.85); *P* < 0.00001). It is suggested that using the TS (TK) principle combined with CWM less than three months or three to six months can both improve the BMD of the lumbar spine and is better than using CWM alone. Two studies reported BMD (lumbar spine) of more than six months [20, 24], and there was high statistical heterogeneity among studies (chi^2^ = 49.53, *P* < 0.00001; *I*^2^ = 98%). We adopted the random-effect model, and meta-analysis showed that there was no significant difference between the experimental and control groups (SMD = 1.36; 95% CI (−1.33, 4.0); *P*=0.32). In general, the meta-analysis showed that different treatment periods combined with CWM were more effective than using CWM alone in improving the BMD of the lumbar spine, with significant differences (SMD = 0.71; 95% CI (0.47, 0.95); *P* < 0.00001) (Figure 3(d)).

#### 3.3.5. BMD (Proximal Femur (Femoral Neck or Total Hip)) of Different Treatment Periods

There were 15 studies which reported BMD (proximal femur (femoral neck or total hip)) of different treatment periods [19, 20, 24, 27, 30, 32, 34, 35, 37, 39, 42, 46, 51–53]. Two studies reported BMD (proximal femur (femoral neck or total hip)) of less than 3 months [27, 37]. High statistical heterogeneity among studies was detected (chi^2^ = 10.41, *P*=0.001; *I*^2^ = 90%); therefore, we adopted the random-effect model, and meta-analysis showed that there was no significant difference between the experimental and control groups (SMD = 0.53; 95% CI (−0.30, 1.36); *P*=0.21). Ten studies reported BMD (proximal femur (femoral neck or total hip)) of three to six months [30, 32, 34, 35, 39, 42, 46, 51–53]. There was high statistical heterogeneity among studies (chi^2^ = 52.61, *P* < 0.00001; *I*^2^ = 83%). We performed sensitivity analysis by removing one trial [35], and low heterogeneity was detected (chi^2^ = 11.68, *P*=0.17; *I*^2^ = 32%). The source of heterogeneity may be related to different treatment methods. So, a random-effect model was adopted. The results showed that using the TS (TK) principle combined with CWM three to six months can improve the BMD of the proximal femur (femoral neck or total hip) and was better than using CWM alone (SMD = 0.69; 95% CI (0.34, 1.04); *P* < 0.00001). Three studies reported BMD of more than six months, and these showed that there was high heterogeneity (chi^2^ = 135.92, *P* < 0.00001; *I*^2^ = 99%) [19, 20, 24]. By removing one study [20], no heterogeneity was detected. The high heterogeneity may result from different treatment methods adopted by these studies. Thus, a random-effect model was adopted, and the results showed that there was no significant difference between the experimental and control groups (SMD = 2.06; 95% CI (0.36, 4.49); *P*=0.10). The combined data showed that different treatment periods combined with CWM were more effective than using CWM alone in improving the BMD of the proximal femur (femoral neck or total hip), with significant differences (SMD = 0.94; 95% CI (0.49, 1.38); *P* < 0.00001) (Figure 3(e)).

#### 3.3.6. Adverse Effects

Eleven studies reported adverse effects [19–21, 30, 33, 34, 36, 37, 39, 41, 53], and there was no statistical heterogeneity among studies (chi^2^ = 4.07, *P*=0.54; *I*^2^ = 0%). Hence, the fixed-effect model was applied to calculate the combined RR and 95% CI as 0.99 (0.65, 1.51), *P*=0.97, indicating no statistically significant difference between the experimental group and the control group. This result suggests that the TS (TK) principle combined with CWM or using CWM alone in the treatment of POP are both safe. Besides, the common adverse effects in the experimental group were gastrointestinal complaints, liver enzyme abnormal, hypertension, joint pain, stomach discomfort, nausea, vomiting, headache, musculoskeletal pain, etc. The adverse effects in the control group included gastrointestinal complaints, liver enzyme abnormal, hypertension, nausea, and vomiting (Figure 3(f)).

#### 3.3.7. TCM Symptom Integral

The TCM symptom integral was established according to the *Clinical Research Guidance of New Chinese Herbal Medicine* [55]. Four studies reported TCM symptom integral [26, 40, 46, 54], and there was low statistical heterogeneity among studies (chi^2^ = 7.49, *P*=0.19; *I*^2^ = 33%). Therefore, the fixed-effect model was applied. The meta-analysis showed that there was a statistically significant difference between the experimental group and the control group (SMD = −1.23; 95% CI (−1.43, −1.02); *P* < 0.00001). This result suggests that the TS (TK) principle combined with CWM in the treatment of POP can significantly improve TCM symptom integral when compared with using CWM alone (Figure 3(g)).

#### 3.3.8. VAS Scores

There were 10 studies which reported VAS scores [21, 23, 25, 31, 34, 40, 43, 48–50]. The result showed that there was high statistical heterogeneity among studies (chi^2^ = 733.80, *P* < 0.00001; *I*^2^ = 99%), so we adopted a random-effect model. The combined data showed that there was a statistically significant difference between the experimental group and the control group (SMD = −3.88; 95% CI (−5.29, −2.46); *P* < 0.00001). This result suggests that the TS (TK) principle combined with CWM in the treatment of POP was more effective than using CWM alone in improving VAS scores (Figure 3(h)).

#### 3.3.9. Publication Bias

**Figure 4:**
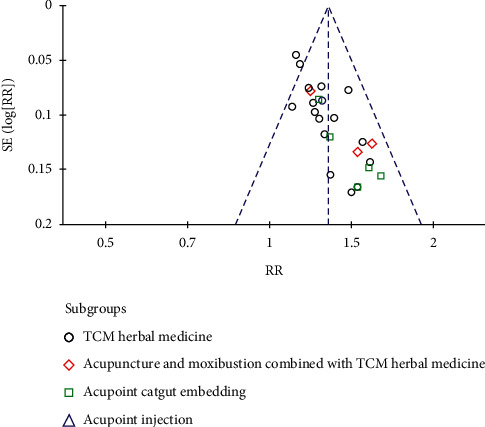
Funnel plot of effectiveness rates of different treatment methods included in the TS (TK) principle.

The funnel plots were generated for studies with data on the effectiveness rates of different treatment methods included in the TS (TK) principle. The results showed that most of the points in the funnel plots were symmetrical. However, two points were outside the 95% CIs, which indicates that there may have been publication bias in our studies and that might influence the results of our analysis, as can be seen in Figure 4.

#### 3.3.10. Quality of Evidence

**Figure 5:**
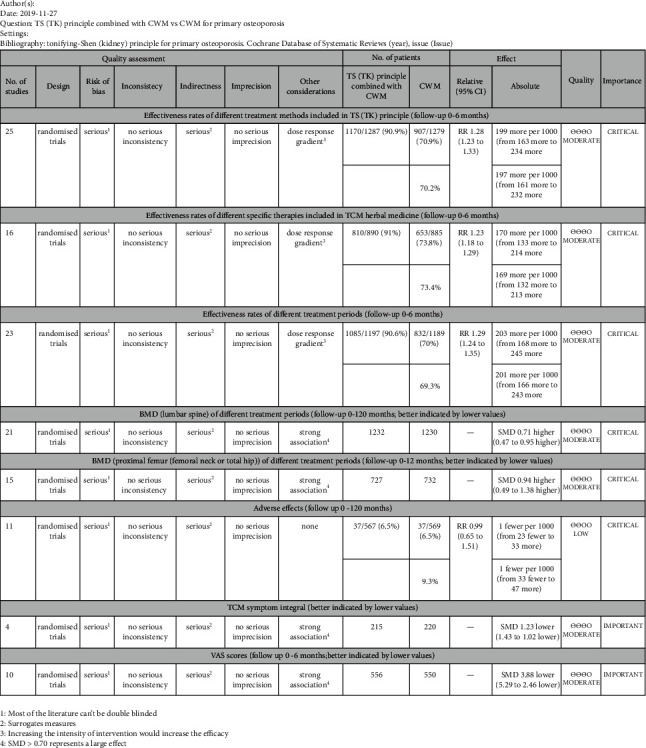
GRADE evidence profile.

There were 8 results for levels of evidence in our study. The detailed GRADE evidence profile of results is shown in Figure 5.

## 4. Discussion

### 4.1. Summary of Main Results

There were 36 included RCTs with 3617 participants in our research. Even though most of the trials had small sample sizes and poor methodological quality, our meta-analysis reached the following results: (1) analysis of the pooled data showed a consistently superior effect of the TS (TK) principle combined with CWM in terms of total effectiveness rates, BMD of the lumbar spine and proximal femur (femoral neck or total hip), TCM symptom integral, and VAS scores when compared to using CWM alone; (2) in terms of adverse effects, the same safety was obtained for the TS (TK) principle combined with CWM or using CWM alone for POP; (3) different treatment methods included in the TS (TK) principle combined with CWM were more effective than using CWM alone in improving effectiveness rates. It should be noted that since the sample size of acupoint injection effectiveness rates was small, the combined RR and 95% CI was reported from the original study, not from the meta-analysis results; (4) in respect of TCM herbal medicine, different specific therapies combined with CWM for POP were more effective than using CWM alone; (5) compared with using CWM alone, the TS (TK) principle combined with CWM was more effective for POP in the aspect of different treatment periods; (6) in the three-to six-month treatment period, the TS (TK) principle combined with CWM for POP in terms of BMD of the lumbar spine and proximal femur (femoral neck or total hip) was better than using CWM alone; (7) according to the guideline of GRADE, the effectiveness rates of different treatment methods included in the TS (TK) principle, effectiveness rates of different specific therapies included in TCM herbal medicine, effectiveness rates of different treatment periods, BMD (lumbar spine) of different treatment periods, BMD (proximal femur (femoral neck or total hip)) of different treatment periods, TCM symptom integral, and VAS scores were moderate level of evidence. The adverse effects were low of evidence.

### 4.2. Analysis of TS (TK) Principle

TCM has been used in a range of medical management and health interventions in China and any other Asian countries for over 2500 years. POP patients are usually seeking TCM treatment, when the therapeutic effect of CWM is unsatisfactory. According to TCM theory, the establishment of a therapeutic principle is based on TCM syndromes, not symptoms. The clinical diagnosis of the TCM syndrome relies on the gathering of clinical information through inspection, auscultation and olfaction, inquiry, and palpation [56]. For POP, the fundamental physiopathological changes of the bone depend on whether Shen (kidney) essence is sufficient or not. Therefore, the TS (TK) principle is the key point to prevent and treat Shen (kidney)-deficiency syndrome of POP [57–60]. On the one hand, the effective mechanisms of pharmaceutical treatment included in the TS (TK) principle on POP have been demonstrated in the voluminous literature. Icariin (ICA), similar to estrogen, has a definite antiosteoporotic effect [61, 62]. Besides, oleanolic acid (OA) and psoralen have been reported to prevent bone loss by inhibiting osteoclast formation [63–65]. On the other hand, in respect of nonpharmaceutical treatment included in the TS (TK) principle, experiments have demonstrated that acupuncture could alleviate osteoporosis by regulating the expression of members in OPG/RANKL, Wnt/*β*-catenin, and MAPK pathways [66]. Acupoint catgut embedding could regulate the hypothalamic-pituitary-ovarian axis to raise the serum *E*_2_ level which would be significant in preventing osteoporosis [67]. Even experiments have shown that acupoint catgut embedding ameliorated the ovariectomization- (OVX-) caused metabonomic changes more effectively than hormone replacement therapy (HRT) with nilestriol [68]. Also, nonpharmaceutical treatment could alleviate related symptoms of osteoporosis and improve the quality of life [43]. Therefore, the TS (TK) principle would be a promising approach for POP, and it can not only improve physiological and biochemical indicators but also alleviate the TCM syndromes [16].

### 4.3. Limitations of Research

However, some limitations in our meta-analysis should be mentioned. (1) Although all of our included studies were RCTs, the methodological quality of them was generally improvable. Most of them failed to describe the blinding methods in detail, allocation, and concealment methods. (2) Among 36 studies, only 8 studies reported follow-up. The longest follow-up period was 120 months, and no further follow-up data were collected. The long-term effect of the TS (TK) principle for POP should be further studied. (3) For POP, fracture incidence should be the most patient-important outcomes. However, no fracture incidence data were collected in our studies. It is necessary to focus on fracture incidence as a patient-important outcome in further studies. (4) The studies included in this analysis were insufficient, especially in terms of subgroup analysis. Thus, potential publication bias probably exists. (5) The high heterogeneity among studies may be related to the different treatment methods, treatment periods, and even the skill level of the practitioners. It is a common problem in the research of TCM therapy. (6) The study for different specific therapies of TCM herbal medicine was insufficient, and further data mining should be carried out.

## 5. Conclusion

In summary, our meta-analysis suggests that using the TS (TK) principle combined with CWM for POP is effective and safe. However, the limitation in the quality and quantity of the included RCTs might weaken the overall reliability of this conclusion. Therefore, large-volume, well-designed RCTs with extensive follow-up are awaited to confirm and update the findings of this analysis.

## Figures and Tables

**Table 1:** The search strategy used in the PubMed database.

Serial number	Search items
#1	Bushen
#2	Tonifying Shen
#3	Bu Shen
#4	Yishen
#5	Nourishing the kidney
#6	Tonifying the kidney
#7	Yi Shen
#8	Tonifying kidney
#9	Nourishing kidney
#10	Nourishing Shen
#11	Reinforcing the kidney
#12	Reinforcing kidney
#13	Reinforcing Shen
#14	Invigorating the kidney
#15	Invigorating kidney
#16	Invigorating Shen
#17	Kidney-reinforcing
#18	Kidney reinforcing
#19	Shen reinforcing
#20	Shen-reinforcing
#21	Kidney-invigorating
#22	Kidney invigorating
#23	Shen-invigorating
#24	Kidney-tonifying
#25	Shen-tonifying
#26	Kidney tonifying
#27	Shen tonifying
#28	Shen invigorating
#29	Invigorating Shen
#30	#1 or #2—#29
#31	Primary osteoporosis
#32	Osteoporosis
#33	Age-related osteoporosis
#34	Age-related osteoporosis
#35	Osteoporosis, senile
#36	Osteoporosis, involutional
#37	Senile osteoporosis
#38	Osteoporosis, age-related
#39	Osteoporosis, age-related
#40	Bone loss, age-related
#41	Age-related bone loss
#42	Age-related bone losses
#43	Bone loss, age-related
#44	Bone losses, age-related
#45	#31 or #32—#44
#46	Randomized controlled trials
#47	Randomized
#48	Randomly
#49	Random
#50	RCTs
#51	#46 or #47—#50
#52	#30 and #45 and #51

**Table 2:** Characteristics of the included studies.

No.	Included studies (author, year)	Age (years)		Participants (experimental group/control group)	Intervention		Outcomes		Adverse effects (experimental group/control)	Treatment periods (months)	Follow-up (months)
		Control group	Experimental group		Control group	Experimental group	Secondary outcomes	Primary outcomes			
1	Zheng et al. [19], 2019	63.9 ± 2.81	63.91 ± 2.86	70/70	Placebo capsules ± calcium carbonate	Bushen Yijing Fang ± calcium carbonate	—	BMD of femoral neck; adverse effects	9/8	36	120
2	Liu and Wang [42], 2016	56.15 ± 6.77	55.86 ± 6.92	62/62	Alendronate sodium tablets ± calcium carbonate D3 tablets	Erxian Bushen decoction ± acupuncture and moxibustion ± alendronate sodium tablets ± calcium carbonate D3 tablets	—	Effectiveness rate; BMD of femoral neck and lumbar spine	—	6	—
3	Xu et al. [43], 2018	63.90 ± 7.59	65.16 ± 6.82	32/31	Calcium carbonate D3	Thunder-fire moxibustion ± calcium carbonate D3	VAS scores	—	—	1	1
4	Zhu et al. [20], 2012	64.9 ± 6.0	65.4 ± 6.3	61/61	Calcium carbonate ± vitamin D	Xian Ling Gu Bao capsules ± calcium carbonate ± vitamin D	—	BMD of femoral neck and lumbar spine; adverse effects	11/11	12	12
5	Zhao and Yan [21], 2018	61.3 ± 4.1	62.1 ± 4.1	50/50	Caltrate ± alpha D3 calciferol	Traditional Chinese medicine prescription ± caltrate ± alpha D3 calciferol	VAS scores	Effectiveness rate; BMD of lumbar spine (L2–4); adverse effects	9/6	6	6
6	Yuan et al. [22], 2014	—	—	40/40	Alendronate ± calcium carbonate D3	Chinese herbal medicine ± alendronate ± calcium carbonate D3	—	Effectiveness rate	—	6	—
7	Huang [23], 2015	65.5 ± 1.6	65.7 ± 1.5	70/70	Calcium carbonate D3 ± alfacalcidol soft capsules	Bushenhuoxuetang ± calcium carbonate D3 ± alfacalcidol soft capsules	VAS scores	Effectiveness rate	—	2	—
8	Wu et al. [24], 2005	56.4 ± 4.6	55.6 ± 4.3	34/34	Caltrate D600	Xian Ling Gu Bao capsules ± caltrate D600	—	BMD of femoral neck and lumbar spine	—	12	—
9	Liu et al. [25], 2011 (3)	62.8 ± 5.9	Liu 2011 (3)^*∗*^: 63.7 ± 3.8; Liu 2011 (3): 61.8 ± 8.3	A: 35/35; B: 35	Calcichew D3 tablets	Liu 2011 (3)^*∗*^: acupoint catgut embedding ± calcichew D3 tablets; Liu 2011 (3): Xianling Gubao capsules ± calcichew D3 tablets	VAS scores	Effectiveness rate; BMD of lumbar spine	—	6	—
10	Lu [47], 2014	62.14 ± 6.34	60.84 ± 6.95	25/22	Calcium carbonate D3	Acupoint catgut embedding ± calcium carbonate D3	—	Effectiveness rate	—	6	—
11	Han et al. [44], 2011	67.39 ± 4. 05	67.42 ± 3. 89	30/30	Caltrate	Shuganwenshentanyushuangjietang ± acupuncture and moxibustion ± caltrate	—	Effectiveness rate	—	6	—
12	Liu et al. [26], 2011 (1)	62.8 ± 5.9	Liu 2011 (1)^*∗*^: 63.7 ± 3.8; Liu 2011 (1): 61.8 ± 8.3	A: 35/35 B: 35	Calcichew D3 tablets	Liu 2011 (1)^*∗*^: acupoint catgut embedding ± calcichew D3 tablets; Liu 2011 (1): Xian Ling Gu Bao capsules ± calcichew D3 tablets	TCM symptom integral	—	0/0/4	6	—
13	Peng et al. [48], 2017	—	—	35/35	Calcichew D3 tablets ± alendronate sodium tablets	Acupoint catgut embedding ± calcichew D3 tablets ± alendronate sodium tablets	VAS scores	Effectiveness rate; BMD of lumbar spine	—	3	—
14	Peng et al. [49], 2018	—	—	45/45	Calcichew D3 tablets ± alendronate sodium tablets	Acupoint catgut embedding ± calcichew D3 tablets ± alendronate sodium tablets	VAS scores	Effectiveness rate; BMD of lumbar spine	—	3	3
15	Zhong et al. [50], 2018	—	—	45/45	Calcichew D3 tablets ± alendronate sodium tablets	Acupoint catgut embedding ± calcichew D3 tablets ± alendronate sodium tablets	VAS scores	Effectiveness rate; BMD of lumbar spine	—	3	—
16	Zou et al. [53], 2018	66.73 ± 3.71	66.27 ± 3.18	60/60	Caltrate D tablets ± alendronate sodium tablets ± calcitriol soft capsules	Acupoint injection ± caltrate D tablets ± alendronate sodium tablets ± calcitriol soft capsules	—	Effectiveness rate; BMD of femoral neck and lumbar spine; adverse effects	0/0	6	—
17	Ma and Fan [45], 2018	68.43 ± 3.68	68.56 ± 3.79	42/42	Calcium carbonate ± vitamin D tablets	Bushenzhuanggutang ± acupuncture and moxibustion ± calcium carbonate ± vitamin D tablets	—	Effectiveness rate	—	3	12
18	Lin [51], 2006 (1)	—	—	24/22	Osteoform capsules	Acupoint catgut embedding ± osteoform capsules	—	BMD of femoral neck	—	6	—
19	Lin [52], 2006 (2)	—	—	20/18	Osteoform capsules	Acupoint catgut embedding ± osteoform capsules	—	BMD of femoral neck	—	6	—
20	Qi [27], 2016	75.42 ± 6.83	76.5 ± 7.28	100/100	Caltrate D	Bushenqianggufang ± caltrate D	—	Effectiveness rate; BMD of femoral neck and lumbar spine	—	3	3
21	Li et al. [28], 2017	75.4 ± 4.9	72.6 ± 5.5	30/30	Calcitriol soft capsules ± caltrate D600	Bushenjianpitang ± calcitriol soft capsules ± caltrate D600	—	Effectiveness rate	—	3	—
22	Hu and Li [29], 2012	—	—	155/145	Caltrate D ± miacalcic	Shangkeyishenjianguwan ± caltrate D ± miacalcic	—	BMD of lumbar spine	—	3	6
23	Liu and Gong [30], 2018	59.88 ± 7.46	60.49 ± 7.25	38/38	Calcitriol soft capsules ± calcium carbonate D3 ± alendronate sodium tablets	Bushenjianpihuoxuefang ± calcitriol soft capsules ± calcium carbonate D3 ± alendronate sodium tablets	—	Effectiveness rate; BMD of femoral neck and lumbar spine; adverse effects	6/4	6	—
24	Chen et al. [31], 2017	66.22 ± 11.3	65.12 ± 12.41	32/28	Salmon calcitonin	Bushenjiangufang ± salmon calcitonin	VAS scores	Effectiveness rate	—	6	—
25	Zou [32], 2016	—	—	31/31	Alendronate sodium tablets ± calcichew D3 tablets	Bushenjiangutang ± alendronate sodium tablets ± calcichew D3 tablets	—	BMD of femoral neck and lumbar spine	—	6	—
26	Han [33], 2013	55.32 ± 1.53	54.08 ± 3.26	28/28	Alfacalcidol soft capsules ± caltrate	Bushenzhuanggutang ± alfacalcidol soft capsules ± caltrate	—	Effectiveness rate; adverse effects	0/0	2	—
27	Huang et al. [34], 2018	—	—	88/88	Alendronate sodium tablets	Bushentang ± alendronate sodium tablets	VAS scores	Effectiveness rate; BMD of femoral neck and lumbar spine; adverse effects	0/0	6	—
28	Huang et al. [35], 2017	/—	—	46/46	Salmon calcitonin	Bushenhuoxuefang ± salmon calcitonin	—	BMD of femoral neck and lumbar spine	—	6	—
29	Song et al. [36], 2015	76.1 ± 3.78	76.4 ± 3.56	90/90	Salmon calcitonin	Bushenhuoxuefang ± salmon calcitonin	—	Effectiveness rate; adverse effects	0/0	12	—
30	Pan and Ding [37], 2014	57. 2 ± 11. 2	56. 9 ± 11. 0	43/43	Oyster shell calcium capsules	Bushenhuoxuefang ± oyster shell calcium capsules	—	Effectiveness rate; BMD of femoral neck and lumbar spine; adverse effects	0/4	3	—
31	Wang [38], 2018	62.25 ± 5.01	62.18 ± 4.58	100/100	Calcium carbonate and vitamin D3 tablets	Bushenhuoxuetang ± calcium carbonate and vitamin D3 tablets	—	Effectiveness rate; BMD of lumbar spine	—	3	—
32	Gui et al. [39], 2017	66.28 ± 8.17	66.19 ± 8.34	30/30	Alendronate sodium tablets ± caltrate D	Bushenyiqihuayutang ± alendronate sodium tablets ± caltrate D	—	Effectiveness rate; BMD of total hip and lumbar spine; adverse effects	4 Feb	6	—
33	Liu et al. [40], 2011 (2)	59.8 ± 8.6	Liu 2011 (2)^*∗*^: 62.5 ± 9.7; Liu 2011 (2): 60.3 ± 10.2	A: 45/45 B: 45	Calcichew D3 tables	Liu 2011 (2)^*∗*^: acupoint catgut embedding ± calcichew D3 tables; Liu 2011 (2): Xian Ling Gu Bao capsules ± calcichew D3 tables	TCM symptom integral; VAS scores	Effectiveness rate; BMD of lumbar spine	—	6	—
34	Ouyang et al. [46], 2011	65.6 ± 6.6	64.4 ± 5.3	25/30	Alendronate	Acupuncture and moxibustion ± alendronate	TCM symptom integral	BMD of lumbar spine and proximal femur	—	6	—
35	Zhou et al. [54], 2016	55 ± 4	56 ± 4	30/30	Salmon calcitonin	Acupoint injection	TCM symptom integral	BMD of lumbar spine	—	2	—
36	Xie et al. [41], 2018	61.00 ± 3.12	59.40 ± 4.12	30/31	Vitamin D chewable tablets ± calcitriol soft capsules	Gushenfang ± vitamin D chewable tablets ± calcitriol soft capsules	—	Effectiveness rate; adverse effects	0/0	3	—

Notes: the experimental group of Liu 2011 (1) was divided into 2 groups, respectively: Liu 2011 (1) and Liu 2011 (1)^*∗*^; the experimental group of Liu 2011 (2) was divided into 2 groups, respectively: Liu 2011 (2) and Liu 2011 (2)^*∗*^; the experimental group of Liu 2011 (3) was divided into 2 groups, respectively: Liu 2011 (3) and Liu 2011 (3)^*∗*^. A: experimental group; B: control group.

**Table 3:** Brief table of risk assessment.

Risk of bias assessment (yes/no/unclear)							
No.	Included studies (first author, year)	Random sequence generation	Allocation concealment	Blinding of participants, personnel, and outcome assessors	Incomplete outcome data	Selective outcome reporting	Other sources of bias
1	Zheng et al. [19], 2019	Randomization number table	Yes	Yes	Yes	No	No
2	Liu and Wang [42], 2016	Randomization number table	Unclear	Unclear	Yes	No	No
3	Xu et al. [43], 2018	Draw opaque envelope randomly	Yes	Yes	Yes	No	No
4	Zhu et al. [20], 2012	Using a computer random number generator	Yes	Yes	Yes	No	No
5	Zhao et al. [21], 2018	Randomization number table	Unclear	Unclear	Yes	No	No
6	Yuan et al. [22], 2014	Randomization number table	Unclear	Unclear	Yes	No	No
7	Huang [23], 2015	Randomization number table	Unclear	Unclear	Yes	No	No
8	Wu et al. [24], 2005	Randomization number table	Unclear	Unclear	Yes	No	No
9	Liu et al. [25], 2011 (3)	Randomization number table	Unclear	Unclear	Yes	No	No
10	Lu [47], 2014	Doll's clinical case random table	Unclear	Unclear	Yes	No	No
11	Han et al. [44], 2011	Randomization number table	Unclear	Unclear	Yes	No	No
12	Liu et al. [26], 2011 (1)	Randomization number table	Unclear	Unclear	Yes	No	No
13	Peng et al. [48], 2017	Randomization number table	Unclear	Unclear	Yes	No	No
14	Peng et al. [49], 2018	Randomization number table	Unclear	Unclear	Yes	No	No
15	Zhong et al. [50], 2018	Randomization number table	Unclear	Unclear	Yes	No	No
16	Zou et al. [53], 2018	Randomization number table	Unclear	Unclear	Yes	No	No
17	Ma et al. [45], 2018	Drawing of lots	Unclear	Unclear	Yes	No	No
18	Lin [51], 2006 (1)	Randomization number table	Unclear	Unclear	Yes	No	No
19	Lin [52], 2006 (2)	Randomization number table	Unclear	Unclear	Yes	No	No
20	Qi [27], 2016	Randomization number table	Unclear	Unclear	Yes	No	No
21	Li et al. [28], 2017	Randomization number table	Unclear	Unclear	Yes	No	No
22	Hu and Li [29], 2012	Randomization number table	Unclear	Unclear	Yes	No	No
23	Liu and Gong [30], 2018	Randomization number table	Unclear	Unclear	Yes	No	No
24	Chen et al. [31], 2017	Coin tossing	Unclear	Unclear	Yes	No	No
25	Zou [32], 2016	Randomization number table	Unclear	Unclear	Yes	No	No
26	Han [33], 2013	Coin tossing	Unclear	Unclear	Yes	No	No
27	Huang et al. [34], 2018	Randomization number table	Unclear	Unclear	Yes	No	No
28	Huang et al. [35], 2017	Randomization number table	Unclear	Unclear	Yes	No	No
29	Song et al. [36], 2015	Draw lots randomly	Unclear	Unclear	Yes	No	No
30	Pan and Ding [37], 2014	Randomization number table	Unclear	Unclear	Yes	No	No
31	Wang [38], 2018	Randomization number table	Unclear	Unclear	Yes	No	No
32	Gui et al. [39], 2017	Randomization number table	Unclear	Unclear	Yes	No	No
33	Liu et al. [40], 2011 (2)	Randomization number table	Unclear	Unclear	Yes	No	No
34	Ouyang et al. [46], 2011	Randomization number table	Unclear	Unclear	Yes	No	No
35	Zhou et al. [54], 2016	Randomization number table	Unclear	Unclear	Yes	No	No
36	Xie et al. [41], 2018	Randomization number table	Unclear	Unclear	Yes	No	No

## Data Availability

The data used to support the findings of this study have been deposited in the following repository: PubMed: https://www.ncbi.nlm.nih.gov/pubmed/; Cochrane Library: https://www.cochranelibrary.com/; EMBASE: https://www.embase.com/; Web of Science: http://webofscience.com/; CNKI: https://www.cnki.net/; VIP: http://www.cqvip.com/; Wanfang: http://www.wanfangdata.com.cn/; CBM: http://www.sinomed.ac.cn/.

## Abbreviations

POP: Primary osteoporosis
OP: Osteoporosis
PMOP: Postmenopausal osteoporosis
CWM: Conventional Western medicines
TCM: Traditional Chinese Medicine
TS (TK) principle: Tonifying-Shen (kidney) principle
RCTs: Randomized clinical controlled trials
BMD: Bone mineral density
VAS: Visual analogue scale
PRISMA: Preferred reporting item for systematic review and meta-analysis
CNKI: China National Knowledge Infrastructure
VIP: Chinese Scientific Journal Database
CBM: China Biology Medicine
MD: Mean difference
SMD: Standardized mean difference
RR: Risk ratio
95% CI: 95% confidence interval
GRADE: Grading of Recommendations Assessment, Development, and Evaluation
ICA: Icariin
OA: Oleanolic acid
OVX: Ovariectomization
HRT: Hormone replacement therapy.
